# Modeling Individual-Level Uncertainty From Missing Data in Multifactorial Breast Cancer Risk Prediction

**DOI:** 10.1200/PO-25-00852

**Published:** 2026-02-06

**Authors:** Bethan L. White, Lorenzo Ficorella, Xin Yang, Kamila Czene, Mikael Eriksson, Per Hall, Stephanie Archer, Marc Tischkowitz, Juliet A. Usher-Smith, Douglas F. Easton, Antonis C. Antoniou

**Affiliations:** ^1^ Department of Public Health and Primary Care, Centre for Cancer Genetic Epidemiology, University of Cambridge, Cambridge, United Kingdom; ^2^ Department of Medical Epidemiology and Biostatistics, Karolinska Institutet, Stockholm, Sweden; ^3^ Department of Oncology, Södersjukhuset, Stockholm, Sweden; ^4^ Primary Care Unit, Department of Public Health and Primary Care, University of Cambridge, Cambridge, United Kingdom; ^5^ Department of Psychology, University of Cambridge, Cambridge, United Kingdom; ^6^ Department of Genomic Medicine, National Institute for Health Research Cambridge Biomedical Research Centre, University of Cambridge, Cambridge, United Kingdom; ^7^ Department of Oncology, Centre for Cancer Genetic Epidemiology, University of Cambridge, Cambridge, United Kingdom

## Abstract

**PURPOSE:**

Multifactorial breast cancer (BC) risk prediction models use a range of predictors to estimate an individual's chance of developing BC. Data on risk factors are often incomplete, and point estimates calculated when data are missing can mask considerable uncertainty. Quantifying this uncertainty is critical for effective risk communication.

**METHODS:**

We used Monte Carlo simulation methods to estimate the distribution of 10-year BC risk for individuals with missing data, using the BOADICEA multifactorial model as an example. Multivariate imputation by chained equations with large representative reference data sets was used to sample missing covariates. We developed a framework for estimating the uncertainty distribution, uncertainty intervals (UIs), and probability of reclassification, which can be applied to any given individual with missing risk factor data. This was applied to estimating individual-level uncertainty distributions and quantifying the probability of reclassification when groups of risk factors are measured, for a range of example women.

**RESULTS:**

Women with limited risk factor data had considerable uncertainty in their estimated BC risk, and 95% UIs spanned all risk categories. This was especially relevant for women classified as moderate-risk, such as those with strong family history or a moderate-risk pathogenic variant. Reclassification probability in this case was as high as 57.5%, with 95% UI of 0.9% to 9.3% for the 10-year risk from age 40 years. Risk certainty improved with additional data collection, particularly genetic information or mammographic density measurement.

**CONCLUSION:**

Our results demonstrate that, in some cases, there is considerable probability of reclassification after collecting missing data. Methodology presented here can identify situations where it would be most beneficial to collect additional information, to enable better informed clinical decision making.

## INTRODUCTION

The average lifetime risk of breast cancer (BC) for women in the United Kingdom is 14%,^1^ with the incidence rising considerably as an individual ages. An individual's personalized BC risk can be estimated using multifactorial risk models that consider their specific risk factors for the disease. This can facilitate risk stratification, and therefore enable early detection and prevention approaches to be targeted toward those at higher risk and most likely to benefit. Available interventions include increased screening frequency, risk-reducing medication, or risk-reducing surgery, which may provide benefits but could have adverse side effects.^2,3^ Risk stratification may also identify lower-risk individuals, which could minimize the adverse effects of surveillance or preventive options, including overdiagnosis, overtreatment, and psychosocial distress.^4^ Targeting screening and prevention more effectively, using personalized BC risk prediction, can also reduce costs to the health care system.^5-7^

**CONTEXT**

**Key Objective**
How can we quantify the uncertainty introduced by missing data at the time of application in personalized multifactorial breast cancer (BC) risk prediction?
**Knowledge Generated**
We developed a statistical framework to estimate the distribution of uncertainty resulting from missing risk factor information in individual risk assessments. This approach enables the calculation of uncertainty intervals around predicted risk, and the probability of risk category reclassification because of missing data.
**Relevance**
Our findings support better-informed clinical decision making by explicitly characterizing the reliability of BC risk estimates in the presence of missing data. This is particularly critical for women whose estimated risks lie near clinical decision thresholds, where a small shift in risk estimate could lead to different risk stratification and clinical management recommendations.

There are several commonly used BC risk assessment tools for personalized risk prediction models,^8-10^ including CanRisk (implementing the BOADICEA model).^11-16^ These tools calculate a point estimate of an individual's BC risk using a range of predictors. In general, risk estimates are given as a percentage risk over a specified time interval, or over a lifetime.

However, risk point estimates are associated with uncertainty. The source of uncertainty considered in this work arises when data that could be included in the model are missing from the risk calculation; this uncertainty could be resolved by collecting all missing data, although this may be difficult because of barriers such as unknown family history, or the cost of measuring certain information.

Quantifying this uncertainty is critical for effective cancer risk communication and better-informed decision making. It can help clinicians decide the extent to which specific additional information would provide more accurate cancer risk prediction, the probability that this information will change an individual's risk category, and whether the improvements in certainty are worth the cost of collecting the additional data. Considerable uncertainty in an individual's risk could indicate that insufficient data have been provided, and suggests that the given risk point estimate should be used with caution.

Currently, none of the commonly used BC risk prediction models^8-10,13^ estimate or communicate uncertainty, even when they allow missing risk factor data. Presenting uncertainty in a way that is credible, trustworthy, accurate, and personally relevant will also be essential to ensure clinical utility and patient engagement.^17-19^

Here, we propose a novel framework for evaluating uncertainty because of missing data in risk prediction, which we have applied to the BOADICEA model.^11^ We used Monte Carlo simulation to calculate the uncertainty distribution of the predicted cancer risk and the probability of reclassification. We illustrate this using practical examples based on different combinations of measured factors. We investigate how measuring additional factors is likely to affect risk point estimates and risk categorization.

## METHODS

### The BOADICEA Model

BOADICEA is a multifactorial breast and ovarian cancer risk prediction model, based on complex segregation analysis, that incorporates explicit cancer family history, lifestyle, hormonal and reproductive factors [questionnaire-based risk factors (QRFs); listed in the Data Supplement, S1.1.1], and mammographic density (MD).^11-16,20^ It also includes a polygenic score (PGS)^21^ and rare pathogenic variants (PVs) in eight BC susceptibility genes. Further details on the BOADICEA model and the underlying disease incidence model are given in the Data Supplement (S1.1). Here, we focus on the methodology for quantifying uncertainty in BC risk prediction.

### Quantification of Uncertainty in the Presence of Missing Data

**FIG 1.**
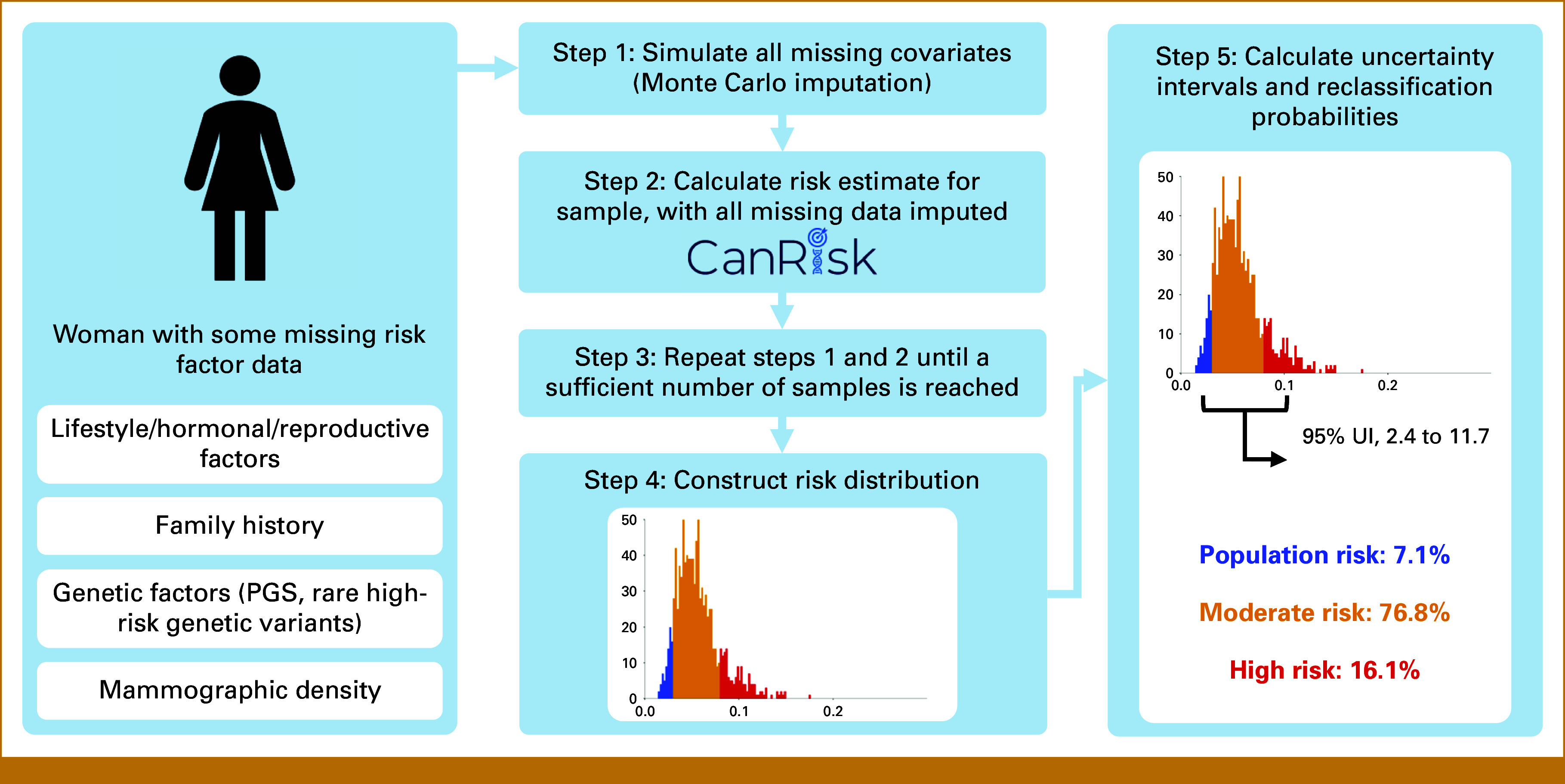
Summary of the process for quantifying uncertainty in personalized BC risk prediction, in the presence of missing data. BC, breast cancer; PGS, polygenic score; UI, uncertainty interval.

To quantify the uncertainty because of missing data, we used multivariate imputation by chained equations (MICE). This approach simulates missing covariates from an approximate joint distribution, conditional on the available data; incorporating randomness into the process generates multiple versions of the complete data for an individual (further details are provided in the Data Supplement, S1.2). Risk was calculated for each imputed sample, thus yielding multiple risk estimates, and those estimates were used to construct the individual's risk distribution (Monte Carlo approximation). We focused on the 10-year absolute BC risk for consistency throughout this work, but risk between age 20 and 80 years is also commonly used for risk classification.^22^ These risk distributions were used to compute 95% uncertainty intervals (UIs) and to estimate the chance of reclassification into a different risk category after measuring unknown risk factors. A 95% UI gives an estimated range of values in which a risk would lie after measuring missing data, with 95% probability. Figure 1 shows an overview of this process.

### Reference Data Sets

We used two reference data sets. UK Biobank is a major biomedical cohort with deidentified genetic data (both PGS and rare PVs), lifestyle data, and other health data from over 500,000 UK participants age 40-69 years at recruitment.^23^ For this analysis, we restricted the data set to women without a previous BC diagnosis at baseline.

KARMA is a cohort of over 70,000 women undergoing mammographic screening as part of the Swedish national screening program. KARMA includes women who were either invited for screening (age 40-74 years) or had a clinical mammography (at any age) at participating sites in Sweden from January 2011 to 2013. In addition to the predictors included in UK Biobank, KARMA also provided measures of MD.^20,24,25^ We restricted the data to women with a MD measurement and without a previous BC diagnosis at baseline.

### Covariate Imputation

In our MICE, QRFs, MD, PGS, and PVs were modeled using logistic regression, multinomial logistic regression, or linear regression, depending on the variable type. MD, BMI, and alcohol use were modeled using linear regression on log-transformed values, conditional on other covariates, to better reflect the population distribution, with an additional probability mass at 0 for alcohol use. PVs were treated as a single variable with nine categories: unaffected, or with a PV in one of the eight major genes considered in the model (under the rare disease assumption, it was assumed that each individual could have a maximum of one PV). This was imputed using multinomial logistic regression. Details of how family history was imputed are given in the Data Supplement (S1.3). To evaluate the imputation process and the underlying assumptions (eg, missing at random, MAR), we compared the distributions of observed and imputed values for four different covariates (Data Supplement, S1.2). Convergence of the MICE imputation was assessed using the Gelman-Rubin statistic^26^ and trace plots of the mean and standard deviation (SD) across iterations for selected covariates.

### Application of MICE Methods

#### Individual-Level Uncertainty Distributions

To demonstrate our approach, we calculated individual risk distributions for women with three different backgrounds: (1) a woman whose mother and sister had BC, both at age 50 years; (2) a woman with a PV in *BRCA1*; and (3) a woman with a PV in *CHEK2*. In all examples, the index woman was assumed to be age 40 years. For each example, we investigated how measuring a PGS (and PV status for the first example) influenced the risk uncertainty distribution. We calculated the probability that, after collecting all missing data, the risk would fall in each risk category, as defined in the NICE guidelines^22^ (near-population risk: 10-year risk <3%; moderate risk: 10-year risk 3%-8%; high risk: 10-year risk >8%), and the 95% UI for the 10-year risk. A thousand (1,000) imputed samples were used to generate each distribution.

#### Probability of Reclassification When Measuring Groups of Risk Factors

To estimate the reclassification probability for an individual when measuring additional factors, we successively imputed additional groups of risk factors to analyze how the distribution of risk estimates changes at each stage, and how this is likely to change an individual's risk category (for details, see the Data Supplement, S1.2). We considered the same example individuals as above. The stepwise reclassification probabilities were visualized using Sankey plots.

Only the risk factors considered measured at each stage of the Sankey diagram were imputed; covariates that were considered unknown at each stage were left as missing when predicting the risk.

#### UIs and Reclassification Probabilities as a Function of PGS

To demonstrate how the variance of risk uncertainty distributions and width of UIs change at higher or lower point estimates of risk, we examined how these change as a function of PGS, with the same given family history and all other information missing. Uncertainty distributions were calculated for individuals with a PGS at the 10th, 20th, 30th, 40th, 50th, 60th, 70th, 80th, and 90th percentiles.

## RESULTS

The cohort characteristics and distributions of the risk factors used in BOADICEA in UK Biobank and the KARMA cohort are shown in the Data Supplement (Tables S1 and S2, respectively). The number of women unaffected by BC at baseline was 264,677 in the UK Biobank and 61,581 in the KARMA cohort. Further details of the two cohorts are provided in the Data Supplement (S2.1).

The Data Supplement (Figs S2-S4) shows diagnostic plots on the performance of the MICE and the validity of the underlying assumptions, with further details in the Data Supplement (S2.2). Imputation diagnostics indicated good chain convergence and similar distributions of imputed and observed values for all covariates.

### Individual-Level Uncertainty Distributions

**FIG 2.**
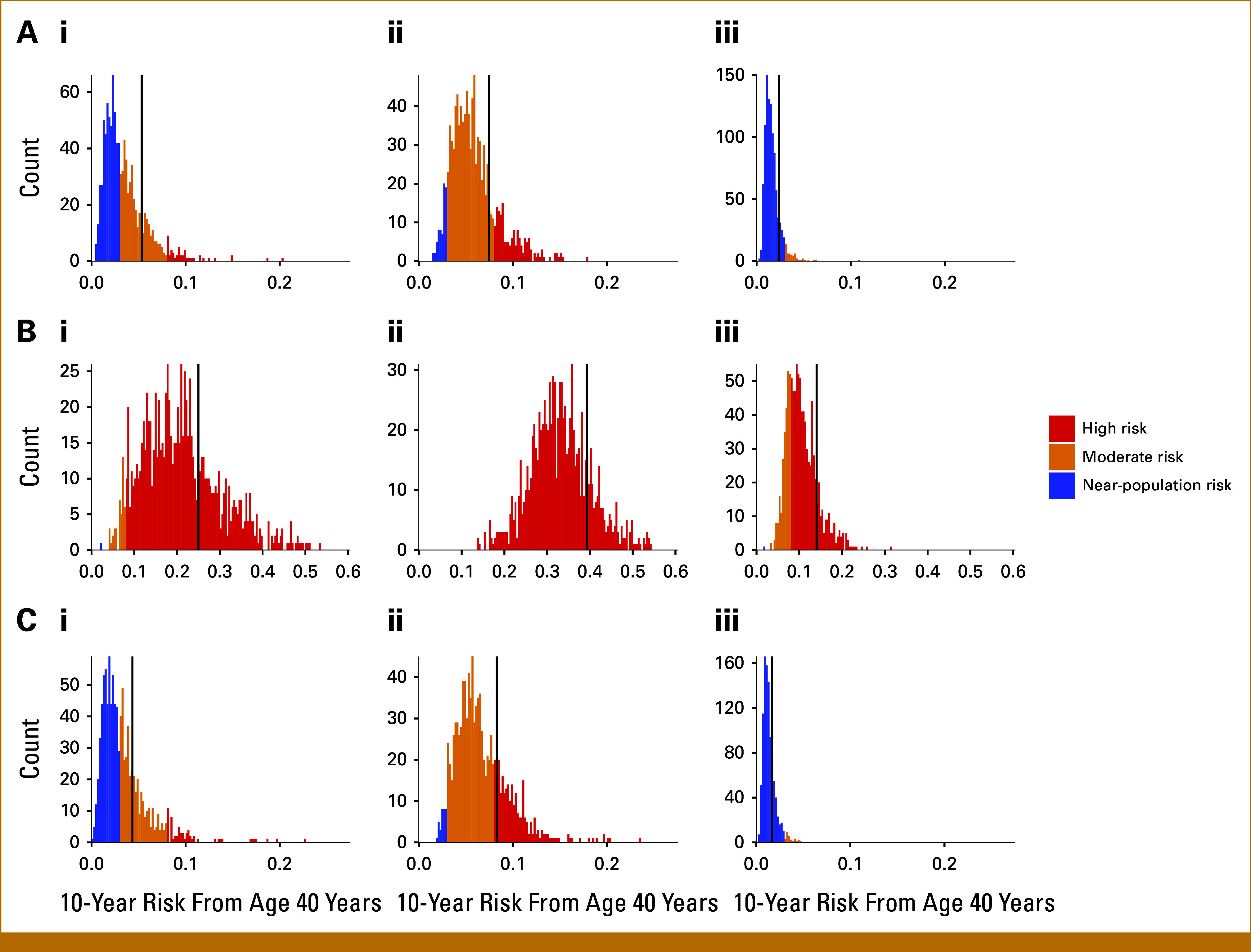
Estimated 10-year BC risk distributions for a 40-year-old woman, with different genetic and family history background; the vertical black line corresponds to the risk point estimate (mean risk over unmeasured covariates) in each scenario. (A) Mother and sister had BC at age 50 years; (B) *BRCA1* PV carrier; (C) *CHEK2* PV carrier. (i) PGS unknown; (ii) PGS = 1.5 SDs above the mean; (iii) PGS = 1.5 SDs below the mean. PGS, polygenic score; PV, pathogenic variant; SD, standard deviation.

Figure 2 shows estimated distributions of 10-year BC risk for the given model examples, first with no additional information, and then with two extreme measured PGS values. For each combination of given information, Table 1 shows the corresponding point estimate (mean risk over unmeasured covariates), the 95% UI, and the proportion of women classified in each NICE risk category.

**TABLE 1.** Point Estimates (mean risk over unmeasured covariates), 95% UIs, and Risk Category Proportions When Risk Factors Are Measured

Individual	Point Estimate, %	Near-Population Risk, %	Moderate Risk, %	High Risk, %	95% UI, %
Background A, no PGS	5.3	52.6	42.5	4.9	0.9 to 9.3
Background A, no major gene PVs, PGS = +1.5 SDs	7.4	7.1	76.8	16.1	2.4 to 11.7
Background A, no major gene PVs, PGS = –1.5 SDs	2.4	94.8	5.1	0.1	0.7 to 3.6
Background B, no PGS	25.0	0.1	4.3	95.6	7.2 to 43.2
Background B, PGS = +1.5 SDs	39.3	0.0	0.0	100	19.0 to 49.9
Background B, PGS = –1.5 SDs	14.0	0.1	25.7	74.2	5.3 to 19.6
Background C, no PGS	4.3	53.9	40.1	6.0	0.7 to 9.8
Background C, PGS = +1.5 SDs	8.3	3.3	70.7	26.0	2.8 to 13.2
Background C, PGS = –1.5 SDs	1.7	97.2	2.8	0.0	0.5 to 3.2

NOTE. Categories defined by NICE guidelines for the 10-year BC risk (near-population risk: <3%; moderate risk: 3%-8%; high risk: >8%). Calculated from risk distributions of the 10-year BC risk of 40-year-old women with different genetic risk factors or family history, assuming (Background A) mother and sister diagnosed with BC at age 50 years; (Background B) *BRCA1* PV carrier; and (Background C) *CHEK2* PV carrier. Corresponding distributions are shown in Figure 2.

Abbreviations: BC, breast cancer; PGS, polygenic score; PV, pathogenic variant; SD, standard deviation; UI, uncertainty interval.

Figure 2A shows the estimated risk distribution for a 40-year-old woman, whose mother and sister were diagnosed with BC (both at age 50 years). With no other information measured (Fig 2, panel Ai), she is classified as at moderate risk (point estimate: 5.3% [95% UI, 0.9% to 9.3%]). When the missing risk factor information becomes measured, there is a 52.6% chance of reclassification to the near-population risk category, and a 4.9% chance of reclassification to the high risk category. With specific measured genetic information, a PGS 1.5 SDs above the mean and no PVs (Fig 2, panel Aii), she remains at moderate risk (point estimate: 7.5% [95% UI, 2.4% to 11.7%]); however, the probability of reclassification to the near-population risk group reduces to 7.1% and to the high risk group increases to 16.1%. With specific measured genetic information, a PGS 1.5 SDs below the mean and no PVs (Fig 2, panel Aiii), she is classified as at near-population risk (point estimate: 2.4%), with a narrower 95% UI (0.7% to 3.6%). The probability of reclassification to the moderate risk group becomes 5.1% and the probability of reclassification to the high risk group reduces to 0.1%.

To demonstrate the impact of missing risk factor information in carriers of PVs in high-risk cancer susceptibility genes, we considered a 40-year-old woman with a *BRCA1* PV (Fig 2B). With no other information (Fig 2, panel Bi), her 10-year risk is estimated as 25.0% (95% UI, 7.2% to 43.2%), with a 4.3% chance of reclassification to moderate risk. With a PGS of 1.5 SDs above the mean (Fig 2, panel Bii), her risk rises to 39.3% with a narrower 95% UI (19.0% to 49.9%) and 0.0% probability of reclassification. With a measured PGS 1.5 SDs below the mean (Fig 2, panel Biii), she remains at high risk (point estimate: 14.1% [95% UI, 5.3% to 19.6%]), with a 25.7% probability of reclassification to moderate risk.

Figure 2C shows the risk distribution for a 40-year-old woman with a *CHEK2* PV. With no other information (Fig 2, panel Ci), she is at moderate risk (point estimate: 4.3% [95% UI, 0.7% to 9.8%]), with 53.9% and 6.0% probabilities of reclassification to near-population and high risk, respectively. With a PGS 1.5 SDs above the mean (Fig 2, panel Cii), she is at high risk (point estimate: 8.3% [95% UI, 2.8% to 13.2%]), with reclassification probabilities of 3.3% to near population and 70.7% to moderate risk. With a PGS 1.5 SDs below the mean (Fig 2, panel Ciii), she is at near-population risk (point estimate: 1.6% [95% UI, 0.5% to 3.2%]), with 2.8% and 0.0% probabilities of reclassification to moderate and high risk, respectively.

### Probability of Reclassification When Measuring Groups of Risk Factors

**FIG 3.**
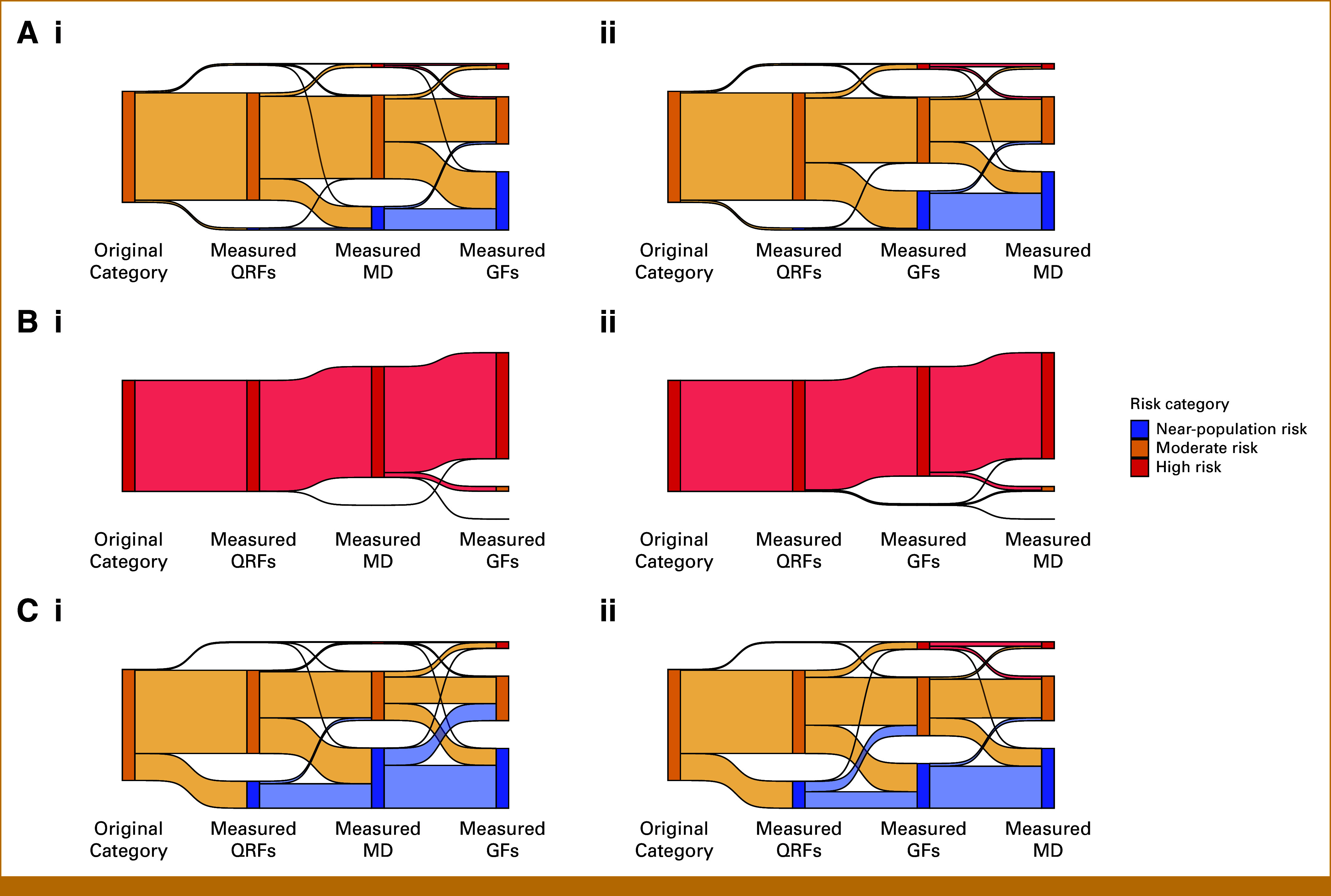
Reclassification when successively measuring QRFs, GFs, and MD, in two different orders, for given individuals. Numbers of individuals moving between categories are shown in the Data Supplement (Table S3). (A) Mother and sister had BC at age 50 years; (B) *BRCA1* PV carrier; (C) *CHEK2* PV carrier. (i) Measuring MD before GFs; (ii) measuring GFs before MD. GFs, genetic factors; MD, mammographic density; PV, pathogenic variant; QRFs, questionnaire-based risk factors.

We investigated the reclassification probabilities when measuring different groups of risk factors successively, to identify risk factors, or groups of risk factors, that have a noticeable impact on the risk reclassification on an individual level (Fig 3). The Data Supplement (Fig S1) shows the risk distributions at each stage, the variation in risk coming from different combinations of missing risk predictors. The Data Supplement (Table S3) gives the number of individuals reclassified from each category at each stage.

Figure 3 (panels Ai and Aii) show reclassification for a woman with BC diagnoses in mother and sister at age 50 years: Figure 3 (panel Ai) shows sequential measurement of QRFs, MD, and genetic factors (GFs; PGS and PVs); Figure 3 (panel Aii) shows measuring QRFs, GFs, and then MD. The measurement order affects reclassification at each stage: adding genetics after QRFs reclassifies 35% to near-population risk and 5% to high risk, whereas measuring MD before genetics provides reclassification probabilities of 20% and 3%, respectively.

Figure 3 (panels Bi and Bii) show results for a *BRCA1* PV carrier. After measuring QRFs, no reclassification occurs. Adding MD leads to 0.1% reclassified to moderate risk, while measuring genetics (PGS) before MD results in 1.3% reclassified.

Figure 3 (panels Ci and Cii) show results for a *CHEK2* PV carrier. With genetics (PGS) and QRFs measured, 34% are reclassified from moderate risk to near population risk, and 9% to high risk. If MD is measured before genetics, the reclassification probabilities are 43% and 2%, respectively.

### UIs and Reclassification Probabilities as a Function of PGS

**FIG 4.**
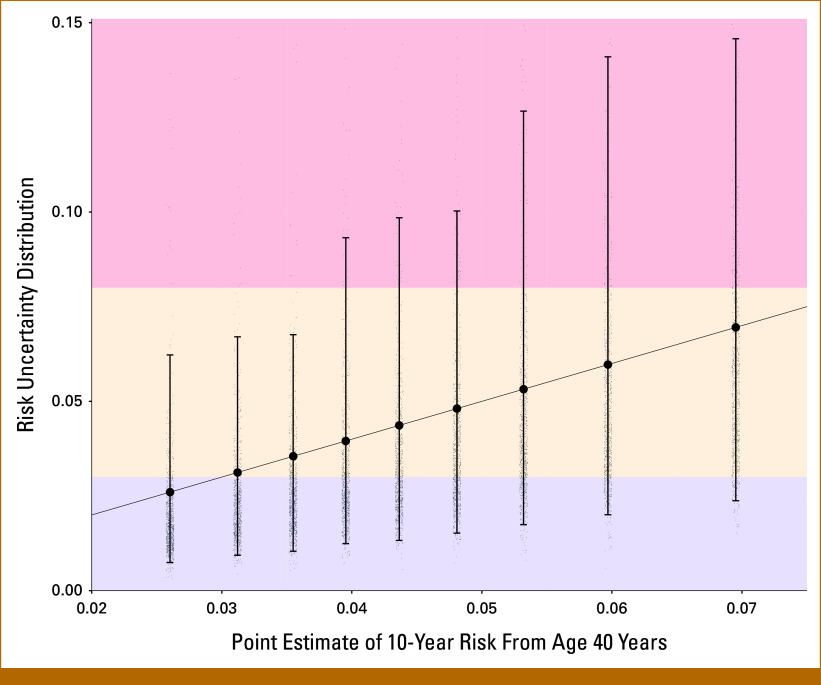
95% UIs and risk distributions for individuals with varying BC risk. Given information: mother and sister had BC at age 50 years, with varied PGS (at the 10th, 20th, 30th, 40th, 50th, 60th, 70th, 80th, and 90th percentiles of the PGS distribution). Each dot represents the predicted 10-year risk, after missing data are imputed. The background shaded regions correspond to the three risk categories as defined in the NICE guidelines on 10-year risk (near-population risk: 10-year risk <3%; moderate risk: 10-year risk 3%-8%; high risk: 10-year risk >8%). PGS, polygenic score; UI, uncertainty interval.

Figure 4 shows risk uncertainty distributions and 95% UIs for a woman whose mother and sister were diagnosed with BC at age 50 years, assuming 9 PGS values (the 10th, 20th, 30th, 40th, 50th, 60th, 70th, 80th, and 90th percentiles), and no information on MD, QRFs, or PVs. The width of the 95% UIs generally increases with higher PGS. Reclassification probabilities for each PGS decile are shown in the Data Supplement (Table S4). For example, at the 10^th^ PGS percentile, the 95% UI is 0.7% to 6.2% with 11.8% total reclassification probability (to moderate or high risk); at the 50th percentile, the 95% UI is 1.3% to 9.8% with a 53.2% reclassification probability (to near-population or high risk); at the 90th percentile, the 95% UI is 2.4% to 14.6%, with a 25.2% reclassification probability (to near-population or high risk).

## DISCUSSION

We developed a novel methodological approach to quantify personalized uncertainty in multifactorial BC risk prediction with missing data, using Monte Carlo simulation and MICE with large reference data sets to estimate BC risk uncertainty distributions. We illustrated the method with clinical examples and varying availability of risk factor data. Our approach quantifies uncertainty by illustrating the distribution of possible risk estimates, and identifying where collecting additional data (MD, PGS, and PVs) could improve risk categorization certainty. This can guide targeted data collection, foster patient trust, and support regulatory assessment of BC risk prediction tools.^27^

The results show that uncertainty in individual BC risk prediction can be high, but can be reduced by measuring additional risk factors, although feasibility must be considered. Some QRFs are easy to collect and should be measured routinely, while others (eg, taking extensive family history) require more time and effort, particularly during a clinical consultation.^28,29^ GFs and MD tend to reduce uncertainty more than QRFs, with BC PGS having the largest impact. However, the costs and practicalities of obtaining genetic or MD data on a routine basis should be considered. The methodology can support case-by-case decisions on which data to collect, aided by tools such as Sankey plots to prioritize information. Clinical consensus recommendations^30^ emphasize incorporating all available information when making risk assessments.

For women with strong BC family history, risk categorization is highly uncertain without additional data. Both genetic and MD information reduce this uncertainty, with genetics having the stronger effect when combined with other risk factors (Figs 3ai-aii). Both can improve classification, helping identify women who remain near-population risk despite strong family history, and should be prioritized in settings where precise estimates guide clinical decisions.

Women with high-risk PVs (eg, *BRCA1*) are rarely reclassified from high risk after full modeling, but missing data still create substantial variation. Collecting additional information before major decisions (eg, medication or surgery) could support better-informed decisions. The same applies to carriers of other high-risk PVs, including *PALB2* or *BRCA2*.^31-35^

Women with moderate-risk PVs (eg, *CHEK2*) have uncertainty spanning all risk categories when no additional information is available. They are key candidates for use of our methodology. Measuring QRFs commonly shifts them to near-population risk (Figs 3ci-cii), and PGS or MD can result in a further shift in category. Similar considerations apply to carriers of other moderate-risk PVs (*ATM*, *BARD1*, *RAD51C*, or *RAD51D*).^36,37^

Women with risk estimates near category boundaries are more likely to be reclassified after measuring missing information. Thus, women near risk thresholds may benefit most from collecting additional data, as shown in Figure 4.

We note that the reclassification probabilities when measuring additional factors have previously been validated against observed incident BC outcomes in both the UK Biobank^38^ and KARMA cohorts.^25^

Currently used BC risk models that allow missing data do not report uncertainty alongside risk point estimates.^8-10,13^ This can lead to overconfidence and obscure the need for further data collection. Riley et al^27^ emphasize the importance of quantifying model uncertainty, which, although different to uncertainty because of missing data, supports the use of UIs in risk prediction. Mathiszig-Lee et al^39^ demonstrated that point estimates alone can mask considerable uncertainty, potentially leading to suboptimal decisions, and that risk distributions improve transparency and can guide data collection. Our approach extends this work by explicitly quantifying and presenting uncertainty from missing data, offering a practical framework for reducing uncertainty through targeted data collection.

This methodology provides a novel approach to quantifying uncertainty in BC risk prediction, but several limitations remain. It is computationally intensive, requiring substantial time for covariate imputation and repeated BOADICEA calculations, making it currently unsuitable for real-time clinical use. Work is ongoing to improve efficiency so uncertainty estimates can be generated quickly. Data set limitations also affect results, including selection or participation biases relative to the UK population, and missing data in key risk factors, including extensive family history (Data Supplement, S3). Importantly, because of the limited sample sizes of participants of non-White ethnicity or non-European ancestry, most of the data used in this work were from White women. Future studies should aim to include more diverse cohorts while accounting for differences in representation within data sets. Recent work has adapted BOADICEA to provide appropriate predictions in ethnically diverse populations.^40^ The methodology presented here could serve as a basis for extending this work to model uncertainty across diverse populations. Our approach also relies on the assumptions underlying the MICE imputation process, but the imputation diagnostics suggest that the missing-at-random assumption is reasonable, with any potential violations having minimal impact on predicted risk uncertainty.

Future work should focus on improving computational efficiency, incorporating detailed family history, adjusting for biases in reference data, and externally validating uncertainty estimates across diverse populations. Other sources of uncertainty, such as model parameter uncertainty and model misspecification, should also be addressed; this will require different methodology.

Clinical use of this work may require guideline changes, and future research should address clinical implementation, such as defining certainty cutoffs before using risk estimates in decision-making. Although this work can inform additional data collection, such efforts must be balanced against cost and accessibility. Several ongoing studies are examining the feasibility of integrating multifactorial cancer risk assessment into existing health care workflows. Initial findings suggest that collecting data for multifactorial cancer risk assessment at the mammographic screening and clinical genetic levels is feasible and acceptable, but requires appropriate infrastructure, resources, and simplified tools for data collection.^41,42^

In conclusion, this framework provides a flexible, generalizable approach to evaluating uncertainty in individualized risk prediction. Identifying when additional information can improve risk stratification will support better-informed clinical decision making. The framework is also adaptable to other cancer risk models, and risk models for other diseases.

## Data Availability

A data sharing statement provided by the authors is available with this article at DOI https://doi.org/10.1200/PO-25-00852. UK Biobank phenotypic and genotypic data are available from UK Biobank. KARMA cohort data are not publicly available because of GDPR regulations, but are available on reasonable request under the Findability, Accessibility, Interoperability, Reproducibility (FAIR) principles, from the Karolinska Institutet through a data access application at: https://karmastudy.org/contact/data-access/. The code used to produce this work can be found at: https://github.com/CCGE-Cambridge/Bethan-UncertaintyModelling. The BOADICEA code is licensed for commercial use by Cambridge Enterprise (University of Cambridge). The code may be shared for research purposes following the completion of a materials transfer agreement with the University of Cambridge.
